# Group Decision Making-Based Fusion for Human Activity Recognition in Body Sensor Networks

**DOI:** 10.3390/s22218225

**Published:** 2022-10-27

**Authors:** Yiming Tian, Jie Zhang, Qi Chen, Shuping Hou, Li Xiao

**Affiliations:** 1 College of Information Engineering, Tianjin University of Commerce, Tianjin 300134, China; 2 School of Engineering, Merz Court, Newcastle University, Newcastle upon Tyne NE1 7RU, UK

**Keywords:** human activity recognition, wearable sensors, selective ensemble learning, sensor data fusion

## Abstract

Ensemble learning systems (ELS) have been widely utilized for human activity recognition (HAR) with multiple homogeneous or heterogeneous sensors. However, traditional ensemble approaches for HAR cannot always work well due to insufficient accuracy and diversity of base classifiers, the absence of ensemble pruning, as well as the inefficiency of the fusion strategy. To overcome these problems, this paper proposes a novel selective ensemble approach with group decision-making (GDM) for decision-level fusion in HAR. As a result, the fusion process in the ELS is transformed into an abstract process that includes individual experts (base classifiers) making decisions with the GDM fusion strategy. Firstly, a set of diverse local base classifiers are constructed through the corresponding mechanism of the base classifier and the sensor. Secondly, the pruning methods and the number of selected base classifiers for the fusion phase are determined by considering the diversity among base classifiers and the accuracy of candidate classifiers. Two ensemble pruning methods are utilized: mixed diversity measure and complementarity measure. Thirdly, component decision information from the selected base classifiers is combined by using the GDM fusion strategy and the recognition results of the HAR approach can be obtained. Experimental results on two public activity recognition datasets (The OPPORTUNITY dataset; Daily and Sports Activity Dataset (DSAD)) suggest that the proposed GDM-based approach outperforms the well-known fusion techniques and other state-of-the-art approaches in the literature.

## 1. Introduction

Recent technological advancements in sensors, wireless communication, and machine learning techniques have driven sensor-based HAR becoming a very active research topic. HAR technology provides an effective way to monitor, analyse and identify one person’s status in different scenes across a variety of human-centred applications and systems [1,2]. Compared with camera-based HAR solutions, sensor-based methods are cheaper and more efficient by using different types of wearable devices (e.g., body-worn inertial sensors, smart glasses, etc.), or smartphones to capture physical activity data. Currently, sensor-based devices have become more accessible in our daily life because they can send data wirelessly to a mobile computing device and greatly reduce users’ awareness and possible discomfort during the process of data collection and recognition. The sensor-based HAR has benefited various fields such as health monitoring [3], assisted living for elderly [4], and sports assessment [5].

Recently, considerable work on HAR has demonstrated the significant role of multi-sensor fusion for wearable activity recognition [6]. According to the data processing level of abstraction, multi-sensor fusion strategies can mainly be categorized into three types: data-level fusion, feature-level fusion, and decision-level fusion [7]. Among these three fusion levels, decision-level fusion provides a unique decision information according to the local decision of multiple sensors, regardless of the sensor properties and parameter settings. The fusion in the decision-level has many advantages such as communication bandwidth saving and allowing the combination of the heterogeneous sensors. However, the most commonly used approaches for this level cannot tackle the uncertainty and ambiguity with complex sensory data [8] and on the knowledge of our scope, there is few literatures emphasis on the study of GDM-based decision-level fusion strategy for HAR issues. 

ELS, as an efficient machine learning technique, has recently become the focus of a new methodology for achieving optimal generalization performance of the HAR task [9]. It overcomes some shortcomings of individual models such as low generalization ability and poor performance due to hyperparameters and data distribution [10]. The principle of ELS is that optimization of the combination of simple models appears more effective than optimizing a single complex model. There are three key factors that are crucial for establishing an optimal MLS: accuracy of individual classifiers, diversity among classifiers, and the applied fusion strategies. Therefore, the proposed ELS in this paper aims to select the optimal ensemble from various information sources of body position and then fuse them based on a GDM-based strategy for HAR. 

The diversity of the base classifiers is a fundamental issue for the success of ELS [11]. Therefore, this paper utilizes two techniques to get multiple base models with diversity. Firstly, in the generation of the base classifiers, variations in training datasets for individual classifiers are considered to improve the diversity among base classifiers. Thus, multiple base classifiers are trained by data from different information sources. Secondly, in the pruning phase of ELS, a mixed diversity measure and a complementarity measure-based pruning methods have been taken into account to optimize the ELS, which seeks to discover a sub-ensemble that improves the performance of the fusion phase. 

Constructing a fusion rule is another crucial factor in ELS, which merges the decision information of individual base classifiers to give the final decision of the ensemble. There are various fusion rules applied in ensemble learning-based HAR, such as majority voting, boosting, bagging, or stacking [12,13,14]. However, the simple information fusion theories are not effective in solving the uncertainty and ambiguity of the information source, especially the types of activity that are not mutually exclusive. This inevitably exists in the decision fusion process and affects the performance of the ELS. Thus, this paper proposes a GDM-based strategy for ensemble learning-based HAR, which is based on the decision information of separate experts from various signal sources. In the proposed combination strategy, each base classifier is regarded as an expert to conduct activity recognition and the GDM strategy is utilized to combine the solutions from the local experts. Each expert is a base classifier model for a part of the multi-sensor fusion problem. Furthermore, the GA (genetic algorithm) algorithm is utilized to acquire the subjective weights of individual experts. 

To summarize, this paper proposes a GDM fusion strategy-based ELS for HAR. Firstly, a decision-level fusion structure is designed for the multi-sensor-based HAR task. Secondly, two techniques are applied to ensure the diversity among the base models: (1) distinctive training data has been taken into account for each base classifier through the relationship between sensors and classifiers; (2) two pruning methods have been proposed to select the best combinations for the fusion phase. Thirdly, a GDM strategy with multiple experts acquiring the decision information from various signal sources is designed to implement the fusion process of the ELS. Two public activity recognition data sets are utilized to verify the effectiveness of the proposed approach. Experimental results show that the proposed group decision making-based ELS approach is more effective than the individual classifiers and approaches based on other fusion strategies.

The rest of this paper is organized as follows. In Section 2, related works about fusion methods for HAR and the diversity of the ELS are introduced. In Section 3, the proposed method is demonstrated in detail. In Section 4, experiments are carried out and results are analysed. Finally, the conclusions and future work are provided in Section 6.

## 2. Related Works 

Considerable research has explored the use of various fusion methods to ELS for HAR. Four decision fusion methods including voting, bagging, boosting, and stacking are utilized to fuse the prediction outputs of multiple classifiers and produce a more accurate classification [15]. The results show that bagging and stacking achieve higher accuracy compared to the baseline method. However, the processing time of these two methods is an obstacle to their specific application. In [16], two fusion schemes are carried out, including the fixed rules and adaptive fuser fusing the results of three mono-modal classifiers for HAR. The results showed that the best performance is achieved by the Product Rule (F1 94.5%) for fixed rules and Random Forest (F1 95.5%) obtains the best performance for adaptive fusing. Ref. [17] proposed a tree-based ensemble learning classifier, XGBoost, to recognize indoor activities. Based on the dataset from 40 volunteers, the proposed XGBoost algorithm outperforms other ensemble learning classifiers and single classifiers and achieves a recognition accuracy of 84.41%. Chen et al. [18] proposed a new method based on the Dempster–Shafer theory to improve HAR by using the fusion of a depth camera and inertial sensors. However, this work ignores the assumption that the hypotheses considered should be exclusive, which is not applicable to HAR. Dong et al. [19] designed a robust and intelligent sensor fusion strategy based on the Dezert–Smarandache theory for HAR. In this framework, the missing data of the involved sensors are ignored without manual interpolation or intervention. 

A large number of works focus on the diversity of the ELS. Chen et al. [20] proposed a novel ensemble extreme learning machine (ELM) algorithm for smartphone-based HAR. The authors utilized Gaussian random projection to initialize the input weights of base ELMs, so the base ELMs in the ensemble have more diversity, which helps to improve the generalization performance of the ensemble system. Cao et al. [21] proposed two new ensemble pruning criteria in the ensemble pruning system for optimizing multi-sensor deployment. Through this work, the new criteria are able to select learners with low error and huge diversity as the final ensemble classifier and the number and type of multi-sensor can be appropriately decided. The kernel density estimation (KDE)-based models are derived from the raw sensor readings so as to build the model pool and the sum of statistical difference and the sum of divergence difference are utilized to select the representative model for a particular activity in [19]. The experiments show that this could effectively guarantee the degree of diversity among base classifiers, which is really important for ensemble fusion. A novel ensemble architecture that fuses three diverse classifiers: layer perception, a random forest, and a support vector machine is proposed in [22]. The ensemble architecture utilizes diversified classifiers upon CNN-extracted features to tackle the WiFi-based HAR task. The results show that the recognition performance of the proposed approach can be improved compared to handcrafted feature extraction or conventional deep learning architectures.

## 3. The Proposed ELS-Based HAR Approach

**Figure 1:**
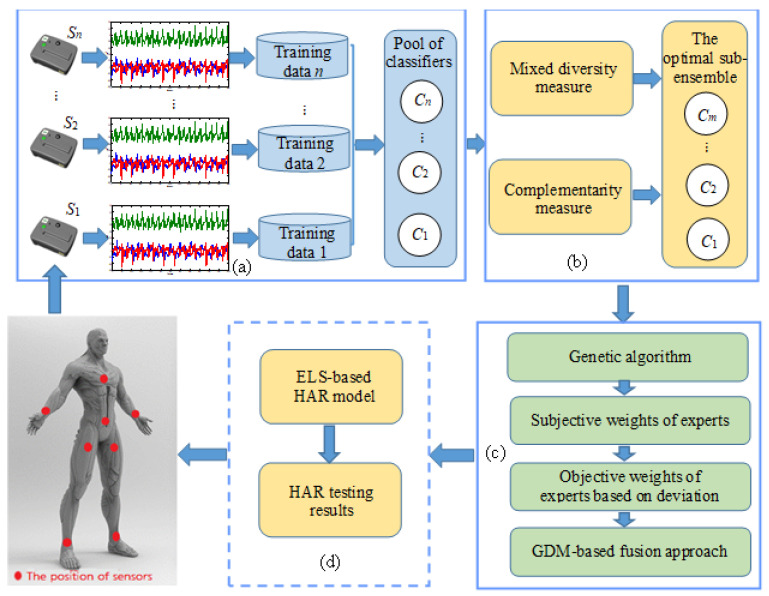
The flowchart of the proposed ELS-based HAR approach. Firstly, in (**a**), diverse base classifiers are trained using training data corresponding to each body position. In (**b**), the optimal sub-ensemble of ELS is selected by taking into account two diversity measures. In (**c**), the GDM-based fusion method is utilized to combine the base classifiers. Finally, in (**d**), the final prediction is produced by the proposed ELS-based HAR model.

The proposed ELS-based HAR approach contains three main phases: (1) generation, (2) pruning, and (3) fusion. The focus of the generation phase is to create a pool of base classifiers. This paper considers the corresponding relationship between sensors and classifiers and the diversity between base classifiers from the data level has also been taken into account. The classifier pool is composed of classifiers corresponding to each body position, which is used in subsequent classifier pruning and fusion phase. In the pruning phase, two new ensemble pruning techniques are applied to select the best classifier from the pool to construct the ELS. The main aim of this phase is to reduce the ensemble scale of ELS and attain better generalization performance and less computational consumption. Finally, in the fusion phase, the base classifiers selected after the pruning phase are considered as experts to recognize activity and obtain independent recognition results, respectively. Due to each expert in a particular position having its own decision information, there will be the problem of information source conflict in the specific HAR. Therefore, this paper proposes a HAR method based on the GDM strategy for fusing the initial results of individual experts, thereby eliminating the information source conflict and improving the accuracy and generation performance. In the following, three phases of the proposed ELS-based HAR approach, including the generation of the pool of base classifiers, selection of base classifiers, and the GDM-based fusion are discussed in detail. The process of our proposed ELS-based HAR approach is illustrated in Figure 1.

### 3.1. Phase One: Base Classifier Generation

The diversity is highlighted as a crucial principle for the design of an ELS, which helps improve the performance of an ensemble by mining salient information with respect to different perspectives. In this phase, to obtain the base classifiers with diversity, a one-to-one relationship between the classifier and sensor is constructed and different classifiers training data are utilized to build the classifier pool. Each classifier is trained by the data collected by sensors at one particular position to acquire the GDM-based decision-level fusion. Compared with data-level and feature-level fusion, decision-level fusion deals with more abstract (higher level) information and arrives at a consensus to improve the overall accuracy, robustness, and generalization. Besides, the ELM [20,23] is utilized in this paper as the base classifier owing to its extremely fast learning speed and the good generalization performance. Moreover, due to the high diversity caused by random parameters of the algorithm, the ELM as an unstable classifier helps build an ensemble learning system with excellent performance.

### 3.2. Phase Two: ELS Pruning 

In the generation phase, base classifiers corresponding to body position are trained for the generation of the base classifiers pool. Thereafter, in the phase of selecting the most appropriate base classifiers to be combined, the diversity and accuracy of the base classifiers are two critical factors in ensemble pruning and can ensure the effectiveness of the ELS. These two important aspects inspire us to research two pruning criteria to discover a set of classifiers (fusion sets) that improve the accuracy of ELS recognition. The first one focuses on the different diversity measures and computes the degree of disagreement of all base classifiers. The second one emphasized the base classifier with the best accuracy and analysed the relationship among them. The two discriminant evaluation functions applied in this paper are shown as follows.

#### 3.2.1. Mixed Diversity Measure

The diversity of classifiers is a key factor for an effective ELS. There must be diversities between the classifiers in the ELS, otherwise, the ensemble results will be the same as those from the individual classifiers. Therefore, it is necessary to select the base classifiers with huge diversity in order to improve the generalization performance and reduce the computational complexity. A new diversity measure is proposed based on the idea of finding the most discriminative component classifier. Therefore, three well-known measures including correlation coefficient *ρ*, *Q* statistical measure, and disagreement measure *Dis* are utilized in the proposed measure. These measurements are defined as follows.

(1) $$\rho_{ij}=\frac{N_{11}N_{00}-N_{10}N_{01}}{\sqrt{\left(N_{11}+N_{10}\right)\left(N_{01}+N_{00}\right)\left(N_{11}+N_{01}\right)\left(N_{10}+N_{00}\right)}}$$

(2) $$Qij=\frac{N_{11}N_{00}-N_{10}N_{01}}{N_{11}N_{00}+N_{10}N_{01}}$$

(3) $${Dis}_{ij}=\frac{N_{10}+N_{01}}{N_{11}+N_{10}+N_{01}+N_{00}}$$

where *N*_11_ is the number of samples that are correctly recognized by both classifiers *C_i_* and *C_j_*. *N*_10_ is the number of samples that are correctly recognized by classifier *C_i_* but misrecognized by *C_j_*. *N*_01_ is the number of samples that are correctly recognized by classifier *C_j_* but misrecognized by *C_i_*. *N*_00_ is the number of samples that are misrecognized by both classifiers *C_i_* and *C_j_*. The range of the diversity measure is (0, 1), which represents the diversity value between the base classifiers *C_i_* and *C_j_*. When there is no diversity between the two base classifiers, the value of diversity measure is 0. The larger the value, the greater the diversity between the two base classifiers. 

According to (1), (2) and (3), another discriminative evaluation function is proposed to measure the discriminative ability between different base classifiers, which is named as the mixed diversity measure. Let *η_i_* denote the average of the sum of the three diversity measures between the base classifier *C*_i_ and all base classifiers and *AV* denote the average diversity value of all base classifiers, that is, the average of the sum of the diverse values of m base classifiers, then they can be calculated as follows:

(4) $$\eta_{i}=\sum_{j=1}^{n}(\rho_{ij}+Q_{ij}+{Dis}_{ij})/n$$

(5) $$AV=\sum_{i=1}^{n}\eta_{i}/n$$

According to Equations (4) and (5), when *η_i_* > *AV*, it indicates that the base classifier *C_i_* has huge diversity with other base classifiers. Then, the base classifier *C_i_* is selected to participate in the final fusion phase and finally *k* base classifiers will be selected according to the value of diversity measure. 

#### 3.2.2. Mixed Diversity Measure

On the other hand, accuracy is another key requirement for a successful ELS. Therefore, the base classifier with the best accuracy in the pool and the relevancy between it and the candidate classifiers is another point that is taken into consideration in this paper. The complementarity measure is utilized to calculate the relevancy between the base classifier with the best accuracy and other candidate classifiers, which is based on the idea of incrementally finding the most complementary component classifier. The base classifier with the optimal performance is first selected and then the base classifier with the greatest complementarity with the currently selected sub-ensemble is selected and added to the sub-ensemble. The process is repeated until sufficient base classifiers are added. The set of base classifiers selected by the complementarity measure not only ensures that the base classifiers have large diversity but also eliminates the base classifiers with similar performance. The process of selecting a base classifier for the complementarity measure is as follows. Assuming that the currently selected sub-ensemble *S_u_* has *u* base classifiers, then the (*u* + 1)th base classifier is selected and add it to *S_u_* to form *S_u + 1_* according to Equation (6):

(6) $$S_{u+1}=\arg\max\sum_{\left(x,y)\in (X,Y\right)}I(Ck(x)=y\;\cap \;E_{Su}(x)\neq y)$$

where *C_k_*(*x*) represents the recognition result of the base classifier *C_k_* for the sample *x*, *E_Su_*(*x*) represents the ensemble result of the sample *x*, and *y* represents the actual category. 

### 3.3. Phase Three: GDM-Based Decision-Level Fusion

After determining a set of classifiers that are more suitable to be fused in phase two, the classifier fusion phase is to exploit the degree of agreement/disagreement among different base classifiers and improve the effectiveness results in the HAR task. In this work, we define the fusion decision phase as an abstract process that includes different experts (base classifiers) who can make individual decisions and the GDM fusion strategy. The final recognition result is produced by experts with the group decision-making strategy, which gives each expert a weight and aggregates the decision-making information of all experts. The weight of experts includes subjective weight and objective weight. The subjective weight is the initial weight that is set based on the factors such as ability level, popularity, and familiarity with decision-making issues. The objective weight is usually determined according to the specific group decision-making issue, the credibility of the expert’s decision and the relationship between the expert’s decision-making results. This paper adopts a common assembly method in group decision-making strategy, specifically as follows:

(7) $$G=\sum_{k=1}^{m}\lambda_{k}U_{k}$$

where *G* = (*g*_1_, *g*_2_,……, *g*_n_)^T^ is the group decision result of fusing all experts, *U_k_* = (*u_k_*_1_, *u_k_*_2_,……, *u_kn_*)^T^ is the decision result made by the *k*th expert, *λ_k_* is the weight of the *k*th expert, which satisfies $\sum {}_{k=1}^{m}\lambda_{k}=1$.

#### 3.3.1. Determination of the Initial Weights of Experts Based on Genetic Algorithm Optimization

The GDM strategy requires a reasonable allocation of the weights of experts. Generally speaking, the experts who have important contributions to the performance of the ELS will be given larger weight values, and vice versa. For this reason, this paper decomposes the weights into initial weights and dynamic weights, which are used as subjective and objective weights of experts respectively. The GA is usually used to obtain the initial weights of multiple groups of experts, and then the dynamic adjustment of weights is realized through the deviation between the individual decision-making results of experts and the group decision-making results in the fusion process. GA [24] has been commonly used to solve an optimization problem, which has the advantage of starting at a population of initial values to find the solution instead of a single initial value. It constantly changes the state of the population by imitating the process of natural selection, and randomly selects individuals as parents to produce the next generation of children. The following operations are required to realize the optimization of the initial weight of the expert by the GA.

(1) Chromosome coding

The chromosome is coded in binary string and the gene chain in the chromosome corresponds to a set of weight vectors of a group of experts. Each bit is represented by a real number 0 or 1. The weight for each expert is represented in each bit of a chromosome. For each combination, the initial weight of the group of experts can be obtained by decoding chromosome *C* by the following equation.

(8) $$W_{i}=\frac{x_{i}}{\sum_{i=1}^{m}x_{i}}$$

where *x*_i_ is the decoding of the *i*th gene *G_i_* of chromosome *C*, *W_i_* is the weight of the *i*th expert corresponding to the *i*th gene *G_i_* and *m* is the number of experts.

(2) Fitness function

In GA, the evaluation of chromosome performance mainly refers to whether the weight of chromosome representatives can achieve the improvement of the accuracy of the activity recognition result. The evaluation of chromosomes is based on their performance and the fitness function of the chromosome is defined as:

(9) $$f=\frac{1}{Y-\sum_{j=1}^{Y}{St}_{j}},\;j=1,2,\cdots ,Y$$

where *Y* is the size of the data set, and $\sum {}_{j=1}^{Y}{St}_{j}$ indicates the number of correctly recognized samples. *St_j_* represents the recognition result obtained by using group decision, it is 1 if the recognition is correct, otherwise it is 0.

(3) Selection operation

The selection operation is performed after evaluating the fitness of the chromosome. This paper utilizes the roulette selection strategy to ensure the convergence of the algorithm while ensuring the performance of the algorithm to implement the global search.

(4) Crossover operation

Crossover operation is an operation method to generate new chromosomes according to swapping some genes of one chromosome with genes of another chromosome. In this paper, the single-point crossover method is selected and a new individual is obtained by swapping the front and back two genes of the preset chromosome gene point.

(5) Mutation operation

The purpose of mutation operation is to improve the search performance of the GA in some local spaces and avoid premature convergency phenomena. The mutation operation used in this paper is the double mutation method that combines overall gene mutation and binary bit mutation. The overall gene mutation is to replace all the information on the gene chain. For example, the overall mutation of gene 001 becomes gene 110 and the mutation on the binary position means that only one bit is taken at a time.

#### 3.3.2. Weight Update Based on Deviation

The subjective weight of the expert reflects the degree of the expert’s contribution to the group decision-making issue, while the objective weight of the expert reflects the hidden information implicit between the individual expert and the expert group. The objective weights of experts are dynamic weights in this paper, which are set based on analysing the deviation between individual expert decisions and group decisions. This paper calculates the weight of experts as follows:

(10) $$\lambda_{k}=\varphi \alpha_{k}+(1-\varphi )\beta_{k}$$

where *α_k_* is the subjective weight of the *k*th expert, *β_k_* is the objective weight of the *k*th expert, and *φ* is the parameter for adjusting the preference of the two weights. 

The fusion approach of GDM should reflect the joint decision-making information of experts. Therefore, on the basis of the subjective weights determined by GA, the objective weights are calculated by using the deviation between the individual expert decision-making results and the group decision-making results, and the objective weights of experts are adjusted to obtain more reasonable values through adjustment. The deviation between the result of the *k*th expert and the group decision-making result can be defined as.

(11) $$r_{k}=\sum_{j=1}^{m}{\left(u_{kj}-g_{j}\right)}^{2},\;k=1,\;2,\;\cdots ,\;m$$

The smaller the deviation, the closer the expert’s decision is to the common will and the expert’ weight should be increased. On the contrary, the expert’s weight should be reduced. Therefore, the weight of each expert can be recalculated as follows based on the degree of deviation.

(12) $$\left\{\begin{array}{l} \lambda_{k}^{i+1}=\lambda_{k}^{i}+\frac{\Delta \lambda }{c},\;r_{k}^{i}<qr_{g}^{i}\\ \lambda_{k}^{i+1}=\lambda_{k}^{i}-\frac{\Delta \lambda }{d},\;r_{k}^{i}>r_{g}^{i} \end{array}\right.$$

where *c* and *d* are the number of experts who made correct and wrong decisions, respectively, Δ*λ* is the adjustment coefficient which is set as 0.05. 

#### 3.3.3. The Fusion Process Based on GDM

**Figure 2:**
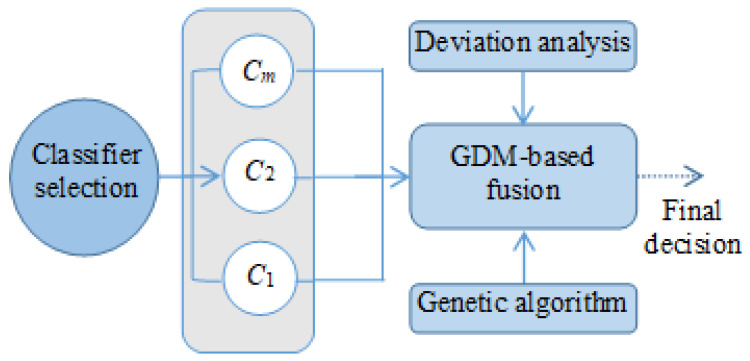
Generalized flowchart of the proposed GDM-based fusion phase.

In this paper, the GDM fusion strategy is applied to fuse the ELS for the activity recognition task. The base classifiers in the ELS are used as the experts in group decision-making, and they are combined at the decision-making level through the GDM fusion strategy. The subjective weight of each expert is determined by the GA and used as the initial weight of the expert. The objective weight of the expert is calculated based on the deviation between the individual decision result of the expert and the group decision result, and the expert weight is dynamically adjusted through the objective weight. Figure 2 shows the generalized flowchart of the fusion phase.

The specific steps of the proposed GDM-based fusion for ELS are as follows:

Step 1 The GA is utilized to give the initial weight of each base classifier (expert) in the ELS.
Step 2 Each expert in the ELS is used to recognize the activity samples and the decision results of the respective experts are obtained which are corresponding to *U_k_* in Equation (7);
Step 3 The group decision result *G* is achieved by the Equation (7), which corresponds to the final recognition result of ELS;
Step 4 Update the expert weight through Equations (10)–(12) according to the result *U_k_* of each expert and the group decision recognition result *G*.
Step 5 Generate the final ensemble learning model and input the testing samples to test its effectiveness.

## 4. Experimental Protocol

### 4.1. Data Sets

In this paper, a selective ensemble learning approach with GDM fusion is proposed for HAR. Two real activity recognition datasets, which were collected from on-body sensors are used to verify the effectiveness of the proposed method. The first dataset is the UCI OPPORTUNITY dataset which contains various sensor nodes on the body positions. The details of this dataset can be found in OPPORTUNITY UCI [25]. In this work, we utilized this dataset to recognize three kinds of daily activities including walking, sitting, and lying, which were performed five times by each of the four subjects. The second dataset is UCI Daily and Sports Activity dataset (DSAD) [26]. This dataset consists of multi-sensor activity data from five body positions collected by the MTx unit. The 19 activities were collected from 8 different subjects in their own style for 5 min in this dataset. In this work, five common daily activities including sitting, standing, lying, walking, and running are recognized to demonstrate the effectiveness of our proposed method. These activities we choose are closely related to the daily activity level and functional health of an individual and can be an indicator for the occurrence of falls. 

### 4.2. Feature Extraction 

Due to a large amount of missing data in the OPPORTUNITY data set, before feature extraction, we need to first preprocess the data set, which includes the processing of missing data and outliers. The processing of missing data is simply to repeat the previous value to replace the missing value. The processing of the outliers is to remove the abnormal spike data of the waveform caused by sensor noise or human factors, and the outliers usually cannot represent the real value of the human activity signal [5,21]. After data preprocessing, a sliding window of 0.5 s is adopted to divide the data into windows with the same length and a 50% overlap between adjacent windows is adopted.

After data preprocessing, the features are extracted from each sensor data for the activities to be recognized on both data sets. Feature extraction takes into account the performance and efficiency requirements of the HAR system. In this work, the maximum, minimum, mean value, root mean square, standard deviation σ, skewness *S*, kurtosis *K* and the signal energy *E* are utilized as feature construction. Some of these features can be expressed as follows:

(13) $$\mathrm{mean}=\frac{1}{N}\sum_{i=1}^{N}a_{i}$$

(14) $$\sigma =\sqrt{\frac{1}{N}\sum_{i=1}^{N}{\left(a_{i}-\mathrm{mean}\right)}^{2}}$$

(15) $$K=\frac{1}{N}\sum_{i=1}^{N}{\left(a_{i}-\mathrm{mean}\right)}^{4}/\sigma^{4}$$

(16) $$S=\frac{1}{N}\sum_{i=1}^{N}{\left(a_{i}-\mathrm{mean}\right)}^{3}/\sigma^{3}$$

(17) $$E=\sum_{i=1}^{N}{\left|a_{i}\right|}^{2}$$

(18) $$RMS=\sqrt{\frac{1}{N}(a_{1}^{2}+a_{2}^{2}\cdots +a_{N}^{2})}$$

where *a_i_* is the sensor data, *i* = 1, 2, …, *N*. *N* is the number of data points. After feature extraction, all features were normalized to the interval [0, 1] to eliminate the impact of the range difference.

### 4.3. Measures of Performance 

The accuracy rate, precision, recall rate and F1 score commonly used in HAR are implemented as indicators to measure the performance of our proposed approach. These measures are defined as:

(19) $$\mathrm{Accuracy}=\frac{TP+TN}{TP+TN+FP+FN}$$

(20) $$\mathrm{precision}=\frac{TP}{TP+FP}$$

(21) $$\mathrm{recall}=\frac{TP}{TP+FN}$$

(22) $$F1=\frac{2\times \;\mathrm{recall}\;\times \;\mathrm{precision}}{\mathrm{recall}\;+\;\mathrm{precision}}$$

where the variables *TP*, *TN*, *FP*, and *FN*, respectively, represent the numbers of true positive, true negative, false positive and false negative outcomes in a given experiment. In addition, F1 evaluation criterion is also considered and is defined as the combination of the precision and the recall. Precision represents the proportion of true positive samples among all samples recognized as positive. Recall represents the proportion of all the positive samples that are recognized as positive samples.

## 5. Experimental Results 

### 5.1. Results on UCI Opportunity Dataset

#### 5.1.1. Effectiveness of the Mixed Diversity Measure and Complementarity Measure

**Figure 3:**
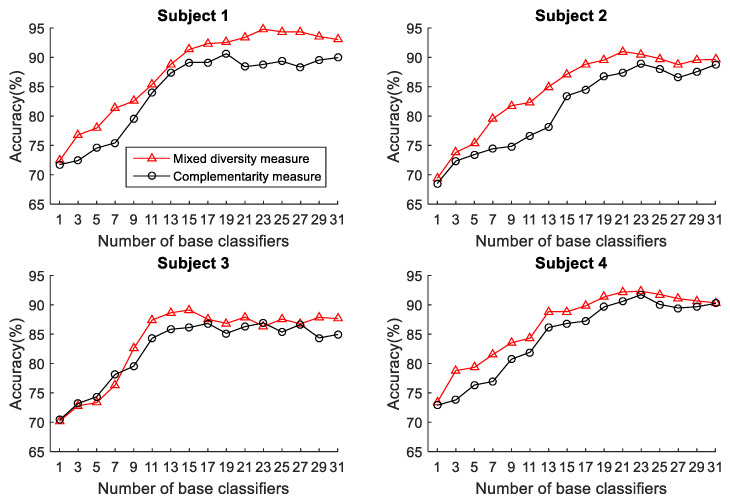
The relationship between the accuracy and the ensemble scale of the ELS in OPPORTUNITY dataset.

To select the more effective method for pre-pruning the initial base classifier pool, the two functions (mixed diversity measure and complementarity measure) are performed on different subjects. The relationship between the accuracy and the ensemble scale of the ELS according to these two functions performed on different subjects is shown in Figure 3. The horizontal axis of Figure 3 represents the ensemble scale. It is worth mentioning that when the basic classifiers are selected, they are arranged in the order chosen by the two algorithms. This helps us specify a more effective pruning method for the OPPORTUNITY dataset and determine the set of base classifiers with excellent performance generated for ELS.

It can be seen from Figure 3 that the ensemble accuracy curves reach a maximum at an intermediate number of base classifiers, and the ensemble classifiers developed using the mixed diversity measure can obtain better performance with the same number of base classifiers than those developed using the complementarity measure. After the maximum, the ensemble accuracy does not rise significantly in fluctuations as the number of base classifiers increases. Therefore, the performance of ELS can be improved after eliminating the redundant base classifiers, in other words, removing some of the base classifiers with small diversity can benefit the ensemble accuracy. Besides, the mixed diversity measure can be employed for pre-pruning base ELMs to achieve better performance. Therefore, the mixed diversity measure is utilized for pre-pruning the OPPORTUNITY dataset. More specifically, the top 8 important sensors selected by the two measures on subjects 1 to 4 are shown in Table 1 and Table 2, respectively, where the sensors selected are arranged in descending order in accordance with the measures. 

The sensors shown in Table 1 and Table 2 contain position and sensor attribute information, where RKN, BACK, RLA, RUA, LUA, LLA, HIP, RWR, LH, and RH represent the right knee, back, right lower arm, right upper arm, left upper arm, left lower arm, hips, right wrist, left hand, and right hand, respectively. Acc, Gyro, and Magn represent the accelerometer, gyroscope, and magnetometer, respectively. According to the experimental results shown in Table 1 and Table 2, we can observe that for specific measure, the choice of sensors for different subjects varies. Whereas for the mixed diversity measure, the RKN/Acc ranks first among the three subjects and the accelerometer occupies most of the important sensors. This shows that due to diverse patterns of personal behaviour, the layout of important sensors is also different, which requires pruning methods to build an activity recognizer with better generalization ability and robustness. For a specific subject, as the mixed diversity measure and complementarity measure have different measurement principles, therefore, the choice of important sensor layout for each subject has different results. 

#### 5.1.2. Comparison with the Individual Classifiers

**Figure 4:**
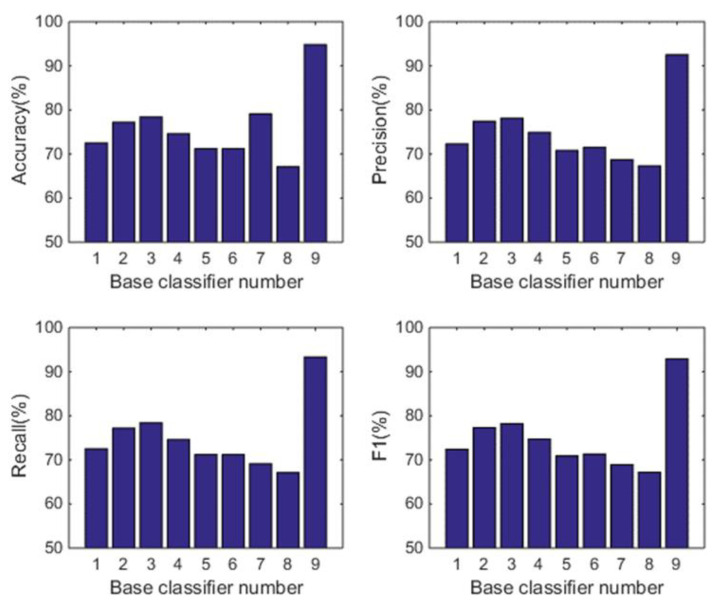
Performance comparison between base classifiers (1–8) and our fused classifier (9) for subject 1 in OPPORTUNITY dataset.

**Figure 5:**
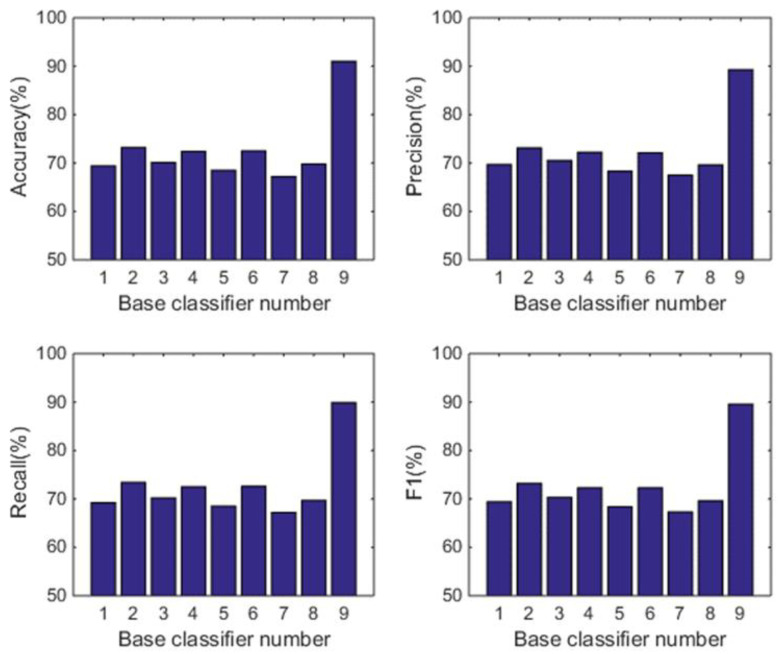
Performance comparison between base classifiers (1–8) and our fused classifier (9) for subject 2 in OPPORTUNITY dataset.

**Figure 6:**
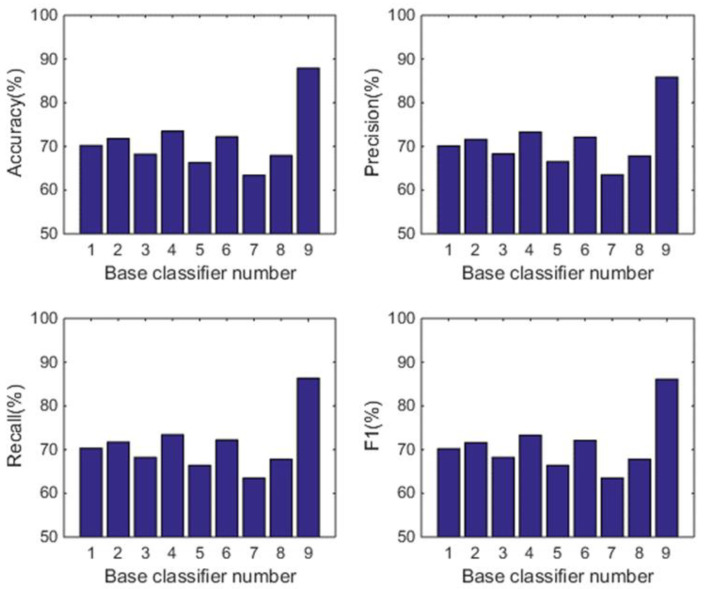
Performance comparison between base classifiers (1–8) and our fused classifier (9) for subject 3 in OPPORTUNITY dataset.

**Figure 7:**
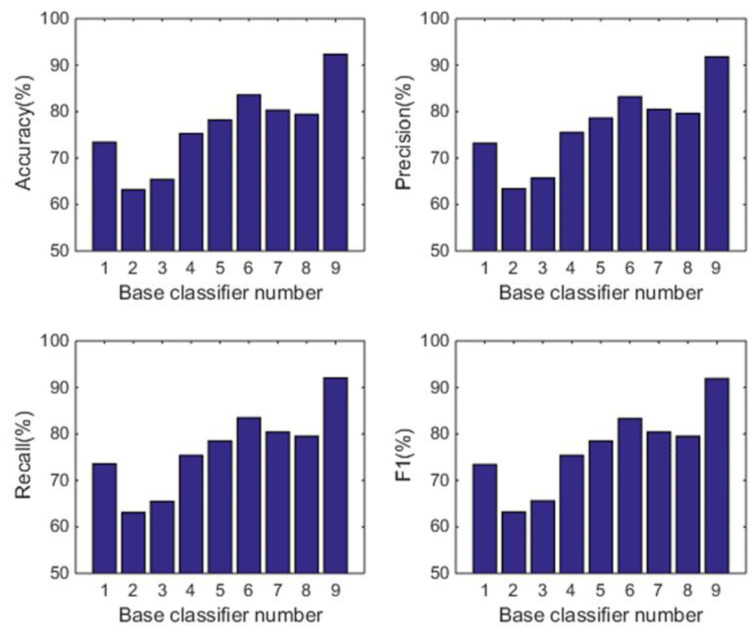
Performance comparison between base classifiers (1–8) and our fused classifier (9) for subject 4 in OPPORTUNITY dataset.

In this subsection, we analyse the power of decision-making fusion in ELS in the situation where the base classifiers are obtained from different body positions. This helps us to understand more clearly the performance improvement of the proposed GDM-based-ensemble approach compared to a single classifier trained with an independent signal source. Based on the results presented in Figure 3, the pruning function chosen here is the mixed diversity measure. The basic classifiers involved in the comparison are selected as the top 8 base classifiers according to the results of the mixed diversity measure shown in Table 1. Figure 4, Figure 5, Figure 6 and Figure 7 show the experimental results where the numbers 1 to 8 represent the first 8 base classifiers respectively and 9 represents our fused classifier. From the experimental results shown in Figure 4, Figure 5, Figure 6 and Figure 7, three points can be concluded. Firstly, it can be found that the proposed approach achieves the optimal performance for all four subjects. Secondly, the performance of the top 8 base classifiers selected for each subject differs greatly whereas the performance of the proposed approach shows stability compared to these individual classifiers. These results further show that the necessity of sensor data fusion to tackle the HAR issue and demonstrate the proposed approach is able to acquire more robust and optimal performance than the individual base classifier. Thirdly, although some of the top base classifiers selected do not show satisfactory performance, for example, the second base classifier of subject 4 and the third base classifier of subject 3, they could achieve an acceptable level of accuracy in an ELS. These show that the proposed approach has always provided more robust performance than other individual classifiers for the four subjects, demonstrating the superiority of our decision-making fusion in ELS. 

#### 5.1.3. Comparison with Other Fusion Strategies and HAR Approaches 

In this subsection, we further evaluate the effectiveness of the proposed GDM-based fusion approach and demonstrate its superiority by comparing it to three fusion methods. In this experiment, four combination strategies including the proposed GDM strategy, GA, weighted average (WA) and majority voting (MV) are used to fuse the base classifiers for the ELS. The idea of the majority vote (MV) is to combine all the results given by each base classifier and select the category with the highest vote. Weighted average (WA) associates the ELS decision with the weights of the base classifiers and the weighted accuracy of each base classifier is utilized as the weight. In GA, the classifiers are combined and the fusion weights are determined using GA to produce the final prediction. 

**Figure 8:**
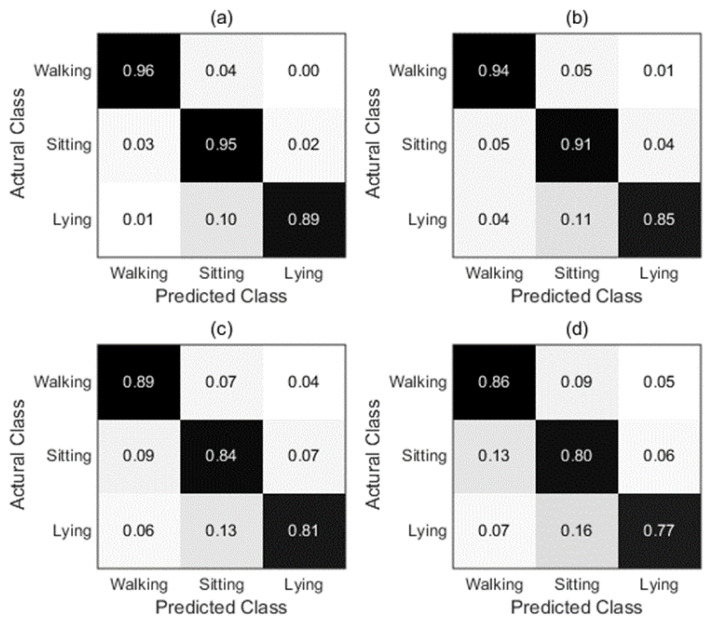
Confusion matrices comparison of different combination strategies for subject 1 in OPPORTUNITY dataset. (**a**) GDM fusion; (**b**) GA; (**c**) WA; (**d**) MV.

Figure 8 shows the corresponding confusion matrices constructed according to different combination strategies for subject 1. The rows and columns of the matrix represent the actual label and the predicted label, respectively. According to Figure 8, more details can be obtained. The recall value of different fusion strategies for each activity is shown in the diagonal elements of the matrix. It is obvious that the recall rate values of the proposed combination strategy are all higher than 90% except for the Lying. However, this is still higher than the recall rate values of other fusion strategies between 77% and 85%. The recall values of the proposed combination strategy are 96% and 95% for walking and sitting, respectively, which are 10% and 15% higher than the lowest recall values in the comparison strategies. In addition, it can be found that there are many misrecognitions between sitting and lying for MV and WA fusion strategies. Nevertheless, the proposed combination strategy effectively reduces the error recognition rate of the two activity modes. 

Table 3 shows the average results of the four fusion strategies for the 4 subjects. The results show that the average accuracies of the four combination strategies are all higher than 80%, except for subject 3 with the MV fusion strategy. The proposed fusion strategy achieves the accuracy of 94.79%, 90.99%, 87.89%, and 92.32% on subjects 1 to 4, respectively. The performance of the GA fusion strategy is slightly inferior, which obtains 92.13%, 88.32%, 84.58%, and 88.52% accuracy for subjects 1 to 4, respectively. MV obtains 82.50%, 84.79%, 79.54%, and 80.84% accuracy for subjects 1 to 4, respectively. According to the above results, it can be found that the proposed combination strategy obtained the highest test accuracy among the four subjects. Besides, the proposed combination strategy acquires higher recognition accuracy than GA in the experiment and this shows the effectiveness of the proposed GDM. Moreover, the MV is the worst fusion strategy compared with the other three fusion strategies. Although the MV strategy is quick and easy, it will introduce some biases caused by the overconfidence of the base classifier. Therefore, if there are many inaccurate classifiers in the ensemble the MV fusion strategy may cause conflicts or misrecognition. The GA optimizes the performance of the fusion classifier by assigning reasonable weights to the base classifier, which reduces the impact of inaccurate base classifiers on the performance of ELS. Our proposed fusion strategy is based on the subjective weight given to experts (base classifiers) by GA and the objective weight is given to the experts (base classifiers) by considering the deviation between the expert and the group decision, which obtains better performance than GA.

Additionally, we also compare the proposed HAR approach with some state-of-the-art HAR approaches, including the DSmt-based method [19], ensemble-extreme learning machine [20], extreme learning machine [21], naive bayes [27], and nearest centroid classifier [28], using the OPPORTUNITY dataset. The results of all the approaches are shown in Table 4. As we can observe in Table 4 our proposed approach outperforms other state-of-the-art approaches except for the DSmt-based method for subject 1. Although DSmt-based method performs better than our GDM-based method for subject 1, our method has achieved optimal performance for subjects 2–4. Besides, for subject 3, our proposed approach and ensemble-extreme learning machine have a relatively similar performance which demonstrates the effectiveness of ELS. Whereas, the recognition accuracy will be very low if a single classifier is considered due to the randomness of the algorithm and parameters. For example, the performances of the extreme learning machine and nearest centroid classifier are greatly affected by their parameters and the centroid of each class. 

### 5.2. Results on UCI DSAD

#### 5.2.1. Effectiveness of the Mixed Diversity Measure and Complementarity Measure 

**Figure 9:**
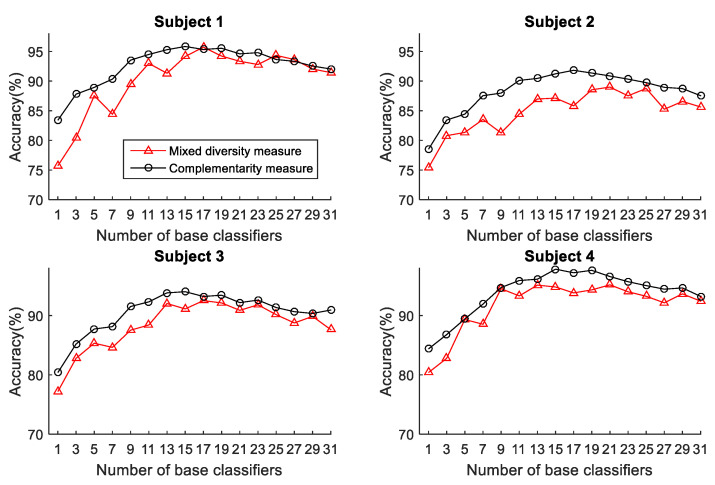
The relationship between the accuracy and the ensemble scale of the ELS in DSAD dataset.

Similarly, we compare the performance of the two pruning methods (mixed diversity measure and complementarity measure) in DSAD. The relationship between the accuracy and the ensemble scale of the ELS according to these two methods performed on subjects 1 to 4 is shown in Figure 9. The base classifiers are still arranged in the order selected by the two methods. It can be seen from Figure 9 that, different from the performance of the complementarity measure in Figure 3, the complementarity measure can achieve better and more robust recognition performance than the mixed diversity measure on the first four subjects in DSAD. Therefore, the complementarity measure will be applied to the following experiment of the DSAD. Besides, as we can see there are few base classifiers in the initial phase of the ensemble, the ELS lacks diversity, which affects its performance. On the contrary, in the later phase of the ensemble, there are a large number of redundant base classifiers, which brings instability to system performance. Additionally, we can also find that the complementarity measure can achieve the best performance when the number of classifiers reaches about 14. Therefore, the ordered ensemble using complementarity measure can pre-prune most redundant base classifiers.

Besides, the top 8 important sensors for complementarity measure on the four subjects are also shown in Table 5. The sensor selection results are arranged in descending order in accordance with the function. The sensors shown in Table 5 contain position and sensor attribute information, where T, RA, LA, RL, LL represent the trunk, right arm, left arm, right leg, and left leg, respectively. xacc, yacc, and zacc represent the three axes of the accelerometer; xgyro, ygyro, and zgyro represent the three axes of the gyroscope and xmag, ymag, and zmag represent the three axes of the magnetometer. According to the experimental results shown in Table 5, it can be found that due to the diversity of individuals, the importance of sensors to different subjects varies and there is no regularity in the selection of important sensors for different subjects. The deployment location and type of sensors will affect the ranking of sensor importance for each subject. This further proves the necessity of the proposed methods for selecting the base classifiers and establishing an ELS based on the optimal subensemble. 

#### 5.2.2. Comparison with the Individual Classifiers

**Figure 10:**
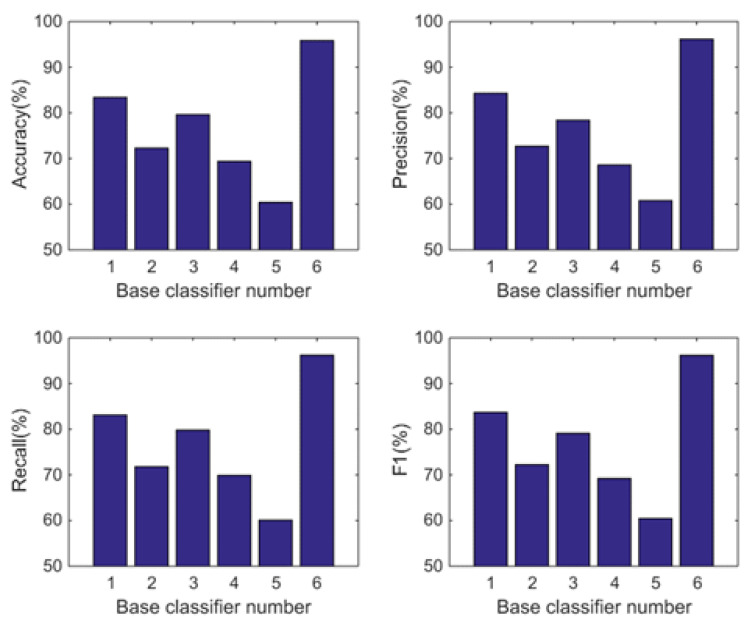
Performance comparison between base classifiers (1–5) and our fused classifier (6) for subject 1 in DSAD dataset.

**Figure 11:**
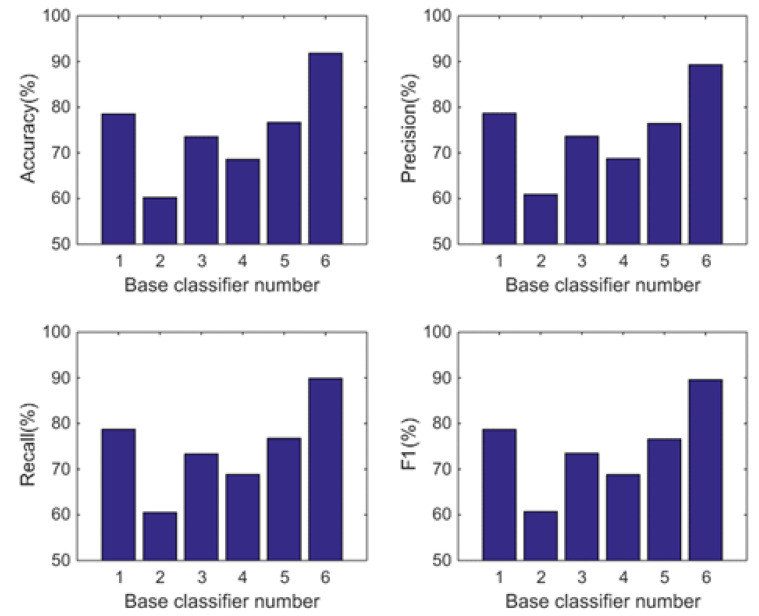
Performance comparison between base classifiers (1–5) and our fused classifier (6) for subject 2 in DSAD dataset.

In this subsection, we also analyse the power of decision-making fusion in ELS in the situation where the base classifiers are obtained from different body positions in DSAD. The performance comparison between the GDM-based-ensemble classifier and the top 5 base classifiers is shown in Figure 10 and Figure 11 where the numbers 1 to 5 represent the first 5 base classifiers respectively and number 6 represents our fused classifier. Based on the results analysed in the previous section, the pruning function chosen here is the complementarity measure. The basic classifiers involved in the comparison are also selected according to the ranking results of the complementarity measure shown in Table 5. From the experimental results shown in Figure 10 and Figure 11, we can observe that (1) Different from the results obtained by the mixed diversity measure (as shown in Figure 3), the complementarity measure first selects the base classifier with the best performance and then adds it to the classifier set according to the principle of maximum complementarity. Therefore, we can see in Figure 10 that the performance of the first base classifier is the best and the performance of the following base classifiers is inferior to the first one. (2) When there are obvious diversities in the recognition performance of the base classifiers (which can also be understood as strong complementarity), the performance of the fusion classifier is improved significantly. Whereas, when the performance between the base classifiers is similar (which can also be understood as weak complementarity), the performance improvement of the fusion classifier is not significant. This further proves the rationality of the maximum complementarity utilized in this paper which considers both the performance of the classifier and the complementarity between the ensemble.

#### 5.2.3. Comparison with Other Fusion Strategies and HAR Approaches

**Figure 12:**
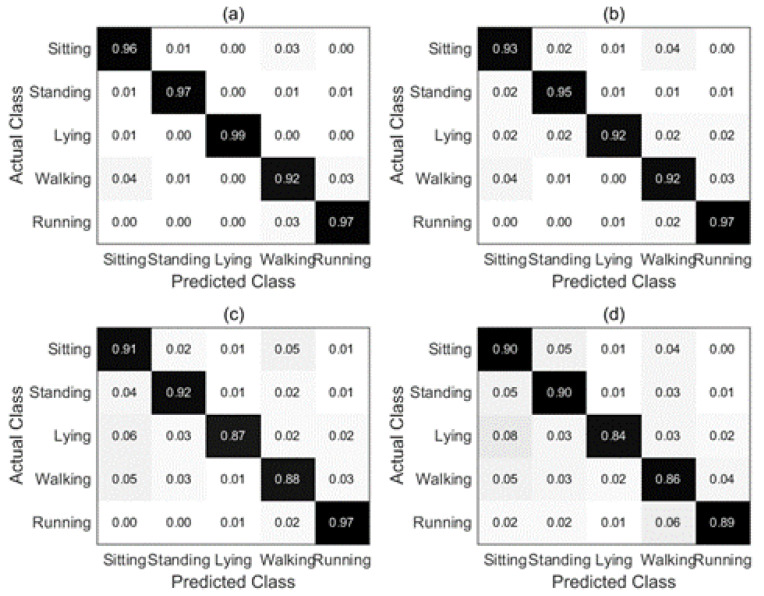
Confusion matrices comparison of different combination strategies for subject 1 in DSAD dataset. (**a**) GDM fusion; (**b**) GA; (**c**) WA; (**d**) MV.

We also evaluate the effectiveness of the proposed GDM-based fusion approach and demonstrate its superiority by comparing it to three fusion methods in DSAD. Figure 12 shows the corresponding confusion matrices constructed with different combination strategies for subject 1 in the DSAD. According to Figure 12, more details can be obtained. Intuitively, the recall rate values of the proposed combination strategy for the five activities are all higher than 90%, lying with the highest recall rate is 99% and walking with the lowest recall rate is 92%. Whereas, the recall rate values of the other combination strategies range from 84% to 92% for lying and walking. Moreover, it can be found that activities sitting and standing are easily recognized by each other. The reason may be that these two activities are relatively similar and the characteristics of the inertial signal are difficult to distinguish. Whereas, the recall rate values of these two activities are significantly improved with the proposed combination strategy. 

Table 6 shows the average results of the four fusion strategies on the first 4 subjects in DSAD. The results show that the proposed fusion strategy achieves the accuracy of the 4 subjects are 95.84%, 91.82%, 94.03%, and 97.76%, respectively, whereas the maximum accuracies for the four subjects of the comparison combination strategies are 91.47%, 87.87%, 92.28%, and 93.47%, respectively. Besides, the recognition accuracies of the MV combination strategy for the four subjects are all lower than 90%, which also demonstrates its shortcoming of bias caused by the overconfidence of the base classifier. Compared with the comparison strategies, the accuracy of the proposed fusion strategy is greatly improved. Especially, in terms of subject 2, the accuracy of the proposed combination strategy is greatly improved to 91.82%, whereas that of the other combination strategies are only 85.42%, 86.04%, and 87.87%, respectively. Similarly, as for subject 1, the accuracy of the proposed combination strategy is greatly improved to 95.84%, while the maximum accuracy of other combination strategies is only 91.47%. These results show that the proposed combination strategy generally acquires optimal performance than the other three comparison strategies.

Besides, the proposed HAR approach is also compared with other state-of-the-art HAR approaches using the DSAD dataset. Table 7 shows the comparison results of the proposed approach with the other traditional methods in references. All the parameters of the mentioned state-of-the-art methods in Table 7 were set based on the literatures, which were not mentioned in detail here. As shown in Table 7, our proposed method has achieved optimal recognition accuracy than other state-of-the-art approaches. Although K-Nearest Neighbours and dRNN show powerful recognition performance and they were not able to properly combine the characteristics of multiple sensors; comparatively, the GDM fusion strategy is proposed to accurately combine the decision information of individual classifiers from multi-sensor data, which proved to be very effective for HAR on DSAD dataset.

## 6. Conclusions

In this paper, an ELS-based decision-level fusion framework with the GDM-fusion strategy is proposed to improve the performance of the HAR task. Multiple diverse base classifiers are constructed with data corresponding to various body positions and two diversity measures are employed to select the best ensemble for the fusion phase. A GDM fusion strategy is proposed to implement the ensemble fusion, and the GA algorithm is used to obtain the initial weights of experts. The GDM fusion strategy is proposed to accurately combine the decision information of individual classifiers from multi-sensor data. The experiments on two public sensor-based HAR datasets (OPPORTUNITY dataset and DSAD) performed in this work demonstrated that there are some differences in terms of important sensor layout depending on the diversity measures considered as well as the personal diversity. Also, the classifier selection can explore the complementary and diverse information provided by different positions, thus improving the performance of ELS. Besides, the performance comparison between the individual classifier and the proposed fusion classifier shows that the effectiveness of multi-sensor data fusion and the GDM fusion strategy contribute to the improvement of performance. Furthermore, the proposed GDM-based ELS achieved excellent performances when compared with other well-known fusion techniques and state-of-the-art approaches in the literature. 

In future work, we will test the performance of the proposed method using more complex activities and attempt to find other techniques for improving the diversity of ELS such as feature diversity, base classifier diversity and other diversity measures. This might enhance the generation performance of ELS and achieve promising results. Also, we may consider using the deep learning methods for adaptive feature learning and the deep learning models are utilized as base classifiers in the ELS. This is because the optimal feature set of each body position might be different. Furthermore, the performance of the proposed ELS-based HAR approach can be enhanced by optimizing the GDM fusion strategy. For example, applying other intelligent algorithms to find the fusion weights is another research direction. 

## Figures and Tables

**Table 1:** Selection results of important sensors in OPPORTUNITY based on mixed diversity measure.

Subject	Selected Sensors
Subject 1	1. RKN/Acc, 2. BACK/Acc, 3. BACK/Magn, 4. RUA/Acc, 5. BACK/Gyro, 6. RLA/Acc, 7. LUA/Gyro, 8 LLA/Acc
Subject 2	1. RKN/Acc, 2. HIP/Acc, 3. RLA/Magn, 4. LUA/Gyro, 5. LLA/Acc, 6. BACK/Gyro, 7. RWR/Acc, 8. RH/Acc
Subject 3	1. RKN/Acc, 2. HIP/Acc, 3. LUA/Acc, 4. LUA/Gyro, 5. RUA/Acc, 6. BACK/Magn, 7. RUA/Gyro, 8. LH/Acc
Subject 4	1. BACK/Acc, 2. RKN/Acc, 3. LUA/Acc, 4. RUA/Acc, 5. LLA/Acc, 6. LUA/Magn, 7. BACK/Acc, 8. HIP/Acc

**Table 2:** Selection results of important sensors in OPPORTUNITY based on complementarity measure.

Subject	Selected Sensors
Subject 1	1. RLA/Acc, 2. RUA/Gyro, 3. LUA/Gyro, 4. RUA/Acc, 5. BACK/Acc, 6. LLA/Magn, 7. LH/Acc, 8 RWR/Acc
Subject 2	1. RUA/Acc, 2. RH/Acc, 3. BACK/Gyro, 4. LUA/Acc, 5. HIP/Acc, 6. BACK/Acc, 7. RUA/Acc, 8. RUA/Magn
Subject 3	1. RKN/Acc, 2. LUA/Acc, 3. BACK/Acc, 4. BACK/Gyro, 5. RLA/Acc, 6. LUA/Gyro, 7. LLA/Acc, 8. HIP/Acc
Subject 4	1. LUA/Acc, 2. LLA/Gyro, 3. BACK/Acc, 4. LLA/Acc, 5. LUA/Gyro, 6. BACK/Gyro, 7. RH/Acc, 8. LUA/ Magn

**Table 3:** The performance comparison of different fusion strategies for 4 subjects in OPPORTUNITY dataset.

Methods	Accuracy			
	Subject 1	Subject 2	Subject 3	Subject 4
MV	82.50%	84.79%	79.54%	80.84%
WA	85.80%	86.87%	82.43%	86.82%
GA	92.13%	88.32%	84.58%	88.52%
Proposed method	94.79%	90.99%	87.89%	92.32%

**Table 4:** Comparison with some state-of-the-art HAR approaches based on UCI OPPORTUNITY dataset.

Methods	Accuracy			
	Subject 1	Subject 2	Subject 3	Subject 4
DSmt-based method [19]	97.14%	88.69%	84.39%	92.62%
Ensemble-extreme learning machine [20]	91.4%	88.43%	87.14%	88.3%
Extreme learning machine [21]	70.56%	71.26%	65.87%	71.54%
Naïve bayes [27]	87.42%	84.01%	82.1%	85.17%
Nearest centroid classifier [28]	83.05%	87.18%	76.47%	81.85%
Proposed method (GDM-based fusion)	94.79%	90.99%	87.89%	93.32%

**Table 5:** Selection results of important sensors in DSAD based on complementarity measure.

Subject	Selected Sensors
Subject 1	1. T/yacc, 2. LA/zgyro, 3. T/xacc, 4. T/zacc, 5. RA/zmag, 6. LL/ ygyro, 7. RL/xmag, 8. RA/yacc
Subject 2	1. T/xacc, 2. RL/xmag, 3. RA/yacc, 4. T/ zgyro, 5. LL/ zacc, 6. LA/ yacc, 7. T/xmag, 8. RL/zacc
Subject 3	1. RA/yacc, 2. T/yacc, 3. LL/ xmag, 4. LA/zacc, 5. T/xmag, 6. T/xacc, 7. RA/zgyro, 8. LL/ xgyro
Subject 4	1. T/zacc, 2. T/xmag, 3. RL/zgyro, 4. T/xgyro, 5. LA/ymag, 6. RA/zmag, 7. RA/xacc, 8. T/xmag

**Table 6:** The performance comparison of different fusion strategies for four subjects in DSAD dataset.

Methods	Accuracy			
	Subject 1	Subject 2	Subject 3	Subject 4
MV	88.64%	85.42%	86.85%	89.72%
WA	90.35%	86.04%	92.28%	91.64%
GA	91.47%	87.87%	91.88%	93.47%
Proposed method	95.84%	91.82%	94.03%	97.76%

**Table 7:** Comparison with some state-of-the-art HAR approaches based on DSAD dataset.

Methods	Accuracy
DSmt-based method [19]	95.1%
pFTA-Learn+ K-Nearest Neighbours [29]	90.2%
KNN + PCA [30]	91.6%
Differential recurrent neural networks [31]	89.6%
K-Nearest Neighbours [32]	86.0%
Proposed method (GDM-based fusion)	95.6%

## Data Availability

The OPPORTUNITY (accessed on 4 January 2021) and DSAD (accessed on 22 March 2022) datasets used in this study are open-source and freely available.
